# Testicular Elastography in Male Infertility: A Systematic Review and Meta-Analysis of Semen Parameters and Azoospermia

**DOI:** 10.3390/jcm15218512

**Published:** 2026-11-02

**Authors:** Federico Falsetti, Angelo Cafarelli, Valentina Maurizi, Michele Marchioni, Luigi Schips, Carlo Giulioni

**Affiliations:** 1 Department of Urology, Casa di Cura Villa Igea, 60127 Ancona, Italy; federicofalsetti92@gmail.com (F.F.); info@angelocafarelli.it (A.C.); 2 Internal Medicine Unit Jesi, Area Vasta 2 ASUR Marche, 60035 Jesi, Italy; valentina.b.maurizi@gmail.com; 3 Department of Urology, Medical School, UniCamillus— Saint Camillus International University of Health and Medical Sciences, 00131 Rome, Italy; michele.marchioni@unicamillus.org (M.M.); luigi.schips@unich.it (L.S.)

**Keywords:** male infertility, shear-wave elastography, testicular stiffness, semen analysis, azoospermia, non-obstructive azoospermia, varicocele, meta-analysis

## Abstract

**Background/Objectives:** Quantitative testicular elastography has been proposed as a non-invasive marker of spermatogenic impairment, but published results differ across semen phenotypes, azoospermia categories, and ultrasound platforms. **Methods:** We performed a PRISMA-conformant systematic review and meta-analysis of observational studies in which quantitative testicular elastography contributed extractable data to at least one meta-analysis. Correlation coefficients were pooled after Fisher-z transformation and back-transformed to r; group contrasts were synthesized as standardized or raw mean differences using random-effects models. SWE/SWV formed the main evidence base, with strain-ratio studies analyzed separately as an ancillary synthesis. Risk of bias was assessed uniformly with the revised JBI tool for analytical cross-sectional studies. **Results:** Eleven studies contributed to the final quantitative synthesis. Mean stiffness was inversely associated with sperm concentration (r = −0.37, 95% CI −0.41 to −0.32, *p* < 0.001; I^2^ = 0%) and motility (r = −0.43, 95% CI −0.58 to −0.24, *p* < 0.001; I^2^ = 8%), whereas morphology was not significantly associated (r = −0.12, 95% CI −0.31 to 0.08, *p* = 0.25). Azoospermic men had greater standardized stiffness than normozoospermic men (SMD 1.695, 95% CI 1.491–1.899; I^2^ = 0%). No evidence of a stiffness difference between NOA and OA was found (SMD 0.47, 95% CI −0.43 to 1.38; I^2^ = 97%). The ancillary strain-ratio retrieval estimate was highly imprecise (SMD 4.10, 95% CI −2.44 to 10.64; I^2^ = 99%). Neither comparison establishes equivalence or excludes a clinically important difference. The stiffness–volume correlation was also highly heterogeneous (I^2^ = 95%). **Conclusions:** Available evidence suggests inverse associations of testicular stiffness with sperm concentration and motility and higher stiffness in azoospermia. Few studies per outcome, a dominant cohort, and technical and statistical heterogeneity limit confidence in the pooled estimates. The NOA–OA, retrieval, and stiffness–volume syntheses are exploratory and cannot support clinical recommendations. Diagnostic thresholds and the value of elastography for individual clinical decisions remain unestablished.

## 1. Introduction

Male infertility accounts for a substantial share of infertility worldwide, yet its diagnosis is rarely straightforward: similar semen patterns may arise from different testicular, genetic, endocrine, obstructive, or systemic disorders [1]. European and North American guidelines therefore combine history and examination with semen analysis, hormonal testing, genetics when indicated, and targeted imaging [2,3]. The 2025 WHO guideline for the prevention, diagnosis, and treatment of infertility provides an additional global framework for clinical care [4]. Semen analysis remains indispensable, but biological variation and the probabilistic nature of WHO reference limits make it a poor stand-alone explanation of an individual patient’s testicular dysfunction [5]. More precise phenotyping has shown how much biological diversity lies behind familiar labels such as oligozoospermia and azoospermia [6]. Reliable interpretation also depends on laboratory standardization, as emphasized in the sixth WHO manual [7]. Reports of declining sperm counts have broadened interest in male reproductive health and potentially modifiable exposures [8], but they do not reduce infertility to a single population trend. It is better understood as a clinical syndrome with multiple causes [9], a view reinforced by the growing number of genetic defects linked to impaired spermatogenesis [10].

Imaging can add information that the ejaculate cannot provide, particularly on testicular volume, parenchymal structure, vascularity, obstruction, and focal disease [11]. Multiparametric scrotal ultrasonography extends this assessment by combining B-mode and Doppler imaging with techniques that probe tissue properties [12]. Shear-wave elastography (SWE), including acoustic radiation force impulse (ARFI)-based techniques such as Virtual Touch Tissue Imaging Quantification (VTIQ; Siemens Medical Solutions USA, Inc., Mountain View, CA, USA), quantifies tissue properties as shear-wave velocity (SWV) or elastic modulus. These measurements are relevant because testicular remodeling may alter tissue mechanics before it is fully captured by semen analysis [13]. The best-developed application is varicocele: Wang et al. found measurable stiffness and volume differences between affected, contralateral, and normal testes [14]. That meta-analysis established biological sensitivity to varicocele-related remodeling, but it did not examine whether elastography tracks semen quality or azoospermia across the wider infertility population. No previous synthesis has combined the main quantitative associations of SWE, ARFI, and VTIQ with semen parameters. We therefore pooled this evidence while keeping modalities and effect measures distinct. A second aim was to identify what must be standardized before testicular elastography can be used as a practical adjunct in male-infertility assessment.

## 2. Materials and Methods

### 2.1. Literature Search

This systematic review was conducted according to the 2020 Preferred Reporting Items for Systematic Reviews and Meta-Analyses (PRISMA) statement [15]. PubMed, Scopus, and Google Scholar were searched from database inception to 28 July 2026. The search strategy included both Medical Subject Headings (MeSH) and free-text terms combined using Boolean operators, as follows: (“Elastography”[Mesh] OR elastograph*[Title/Abstract] OR sonoelastograph*[Title/Abstract] OR sono-elastograph*[Title/Abstract] OR elastosonograph*[Title/Abstract] OR “elasticity imaging”[Title/Abstract] OR “tissue elasticity imaging”[Title/Abstract] OR “strain imaging”[Title/Abstract] OR “strain elastography”[Title/Abstract] OR “strain ratio”[Title/Abstract] OR “real time elastography”[Title/Abstract] OR “real-time elastography”[Title/Abstract] OR “compression elastography”[Title/Abstract] OR “shear wave elastography”[Title/Abstract] OR “shear-wave elastography”[Title/Abstract] OR “shear wave imaging”[Title/Abstract] OR “shear-wave imaging”[Title/Abstract] OR “shear wave velocity”[Title/Abstract] OR “shear-wave velocity”[Title/Abstract] OR “point shear wave elastography”[Title/Abstract] OR “point shear-wave elastography”[Title/Abstract] OR pSWE[Title/Abstract] OR “two dimensional shear wave elastography”[Title/Abstract] OR “two-dimensional shear-wave elastography”[Title/Abstract] OR “2D shear wave elastography”[Title/Abstract] OR “2D-SWE”[Title/Abstract] OR SWE[Title/Abstract] OR “acoustic radiation force impulse”[Title/Abstract] OR “acoustic radiation force impulse imaging”[Title/Abstract] OR ARFI[Title/Abstract] OR “virtual touch tissue imaging”[Title/Abstract] OR “virtual touch tissue quantification”[Title/Abstract] OR VTIQ[Title/Abstract] OR “supersonic shear imaging”[Title/Abstract] OR “tissue stiffness”[Title/Abstract] OR “elastic modulus”[Title/Abstract] OR elastogram*[Title/Abstract]) AND (“Testis”[Mesh] OR testis[Title/Abstract] OR testes[Title/Abstract] OR testicle*[Title/Abstract] OR testicular[Title/Abstract] OR intratesticular[Title/Abstract] OR “testicular tissue”[Title/Abstract] OR “testicular parenchyma”[Title/Abstract]). No restrictions on publication date were applied. The review protocol was registered in PROSPERO (registration number: CRD420261485639).

### 2.2. Eligibility Criteria

The PICOS (Population, Intervention, Comparison, Outcomes, and Study Type) framework was used to define the eligibility criteria and address the clinical question. Population: Adult men investigated for male infertility or with a defined infertility-related condition, including abnormal semen parameters, azoospermia, non-obstructive azoospermia (NOA), obstructive azoospermia (OA), and infertility-associated varicocele. Index test/exposure: Quantitative testicular elastography, including SWE, SWV, elasticity/stiffness measurements, and quantitative strain ratios. SWE/SWV formed the main evidence base. Strain-ratio studies were retained in a separate ancillary synthesis of sperm retrieval and were not pooled with SWE. Comparison: Normozoospermic or normal-semen controls, OA patients, or successful sperm-retrieval groups, according to the specific comparison; no separate control group was required for within-cohort correlation analyses. Outcomes: Associations between elastographic parameters and sperm concentration, motility, morphology, and testicular volume; differences in elastographic parameters between azoospermic and normozoospermic men, abnormal/oligospermic and normospermic men, NOA and OA, and successful versus failed sperm-retrieval groups. Study Type: Randomized controlled trials and prospective or retrospective observational studies were considered eligible.

### 2.3. Study Screening and Selection

Two independent authors screened all retrieved records using Covidence Systematic Review Management^®^ (Veritas Health Innovation, Melbourne, Australia), with disagreements resolved by a third author. Studies were selected according to the predefined PICOS eligibility criteria. Reviews, conference abstracts, letters to the editor, case reports, non-English-language articles, and editorials were also excluded. Full-text articles were assessed whenever the title and abstract indicated potential relevance to the study objectives. In addition, the reference lists of all articles undergoing full-text assessment were manually screened to identify further potentially eligible studies.

### 2.4. Statistical Analysis

Continuous outcomes were pooled using inverse-variance random-effects models and expressed as standardized mean differences (SMDs) when scales differed and mean differences (MDs) when a common scale was retained, with 95% CIs and *p* values. Pearson and Spearman correlation coefficients assessing the same clinical construct were pooled on the Fisher-z scale, using z = atanh(r) and SE = 1/√(n − 3). Pooled estimates and confidence limits were back-transformed using r = tanh(z) and are reported as correlation coefficients in the text and figure legends. The native Review Manager (RevMan) 5.4.1 (The Cochrane Collaboration, Copenhagen, Denmark) exports were retained. In their selected ratio-format display, the headings log[Odds Ratio] and Odds Ratio represent z and exp(z), respectively, rather than clinical odds ratios. The plotted reference value of 1 corresponds to r = 0; values below and above 1 indicate inverse and positive associations. Meta-analysis was undertaken when at least two studies addressed a comparable outcome using compatible effect measures [16]. All analyses were two-tailed, with statistical significance defined as *p* < 0.05.

Heterogeneity was quantified using I^2^ statistics, with I^2^ > 50% considered substantial. When studies reported medians, ranges, and sample sizes instead of means and standard deviations, the latter were estimated using the method described by Hozo et al. [17]. Meta-analysis was performed using Review Manager (RevMan), version 5.4.1 (The Cochrane Collaboration, Copenhagen, Denmark). Numerical verification used Python 3 (Python Software Foundation, Beaverton, OR, USA) and the same inverse-variance formulas. Risk of bias was assessed using the revised JBI critical appraisal tool for analytical cross-sectional studies [18]. This choice was made because the effects entering the meta-analyses were cross-sectional associations or contemporaneous group contrasts even when the parent publication had a broader prospective or diagnostic design. Overall judgments were derived from the pattern and importance of domain concerns rather than from a numerical quality score. The statistical unit followed the source estimate. Semen-correlation inputs used numbers of men, with bilateral averages in Cui et al. Some secondary syntheses included testis-level observations, identified in Table 1 and the relevant Results. Mixed totals are therefore described as analytical observations rather than independent patients. Paired-testis covariance could not be reconstructed from the aggregate reports, and random-effects pooling does not correct this within-study dependence. Unaddressed dependence may overweight testis-level contributions and make the reported CIs and *p* values overly precise; these are nominal results for the affected exploratory syntheses.

## 3. Results

### 3.1. Study Screening

**Figure 1:**
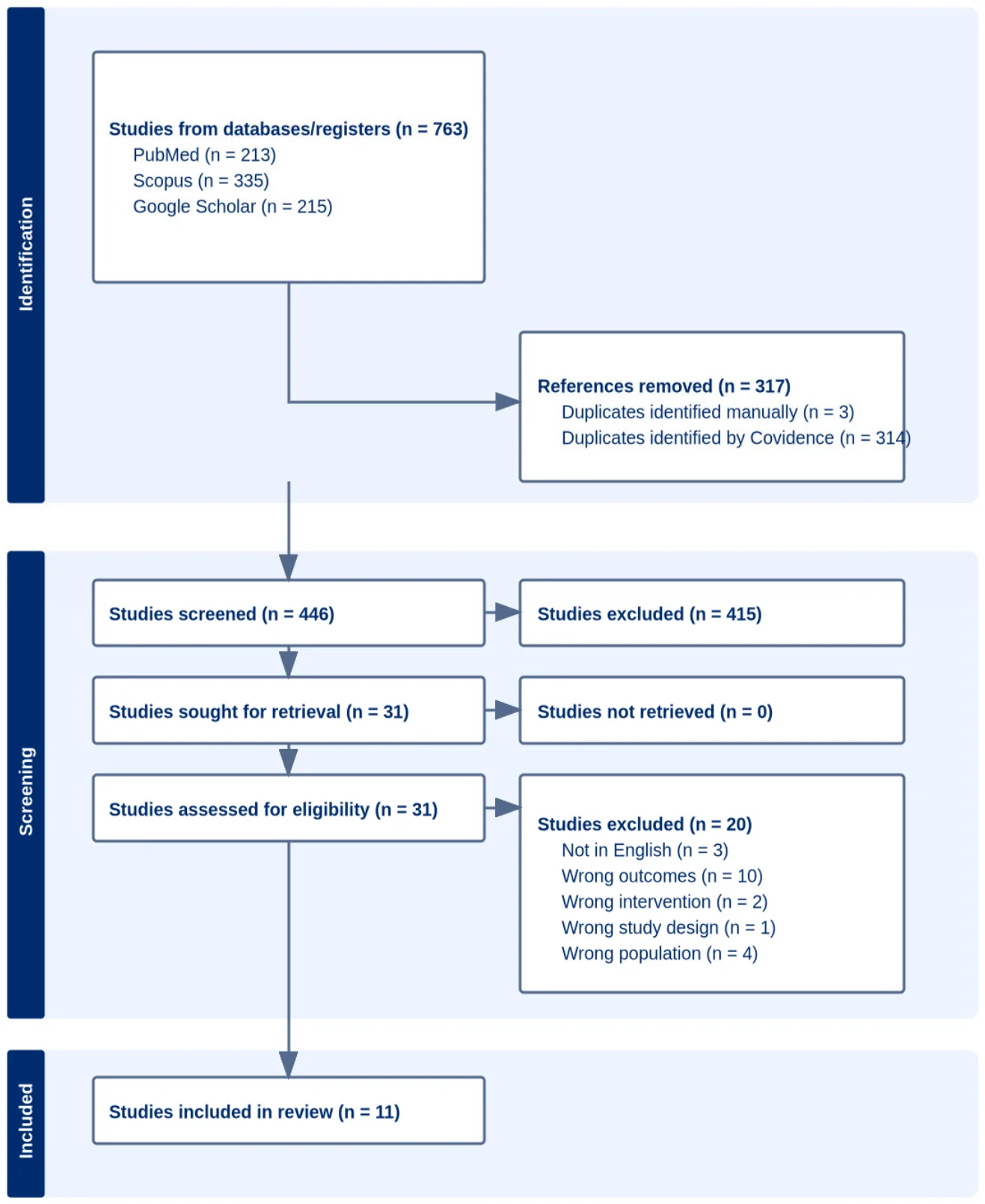
PRISMA 2020 flow diagram.

The search identified 763 records; 317 duplicates were removed and 446 records underwent title and abstract screening. Of these, 415 were excluded. Thirty-one full-text articles were assessed, 20 were excluded, and 11 studies were included in the systematic review and meta-analysis [19,20,21,22,23,24,25,26,27,28,29]. The PRISMA 2020 flow diagram is shown in Figure 1.

### 3.2. Study Characteristics

The clinical populations reflected different components of the male-infertility spectrum. Abdelwahab et al. [19] evaluated testicular stiffness in varicocele-associated abnormal semen parameters; Cui et al. [20] provided the largest cohort and contributed to analyses of sperm concentration, azoospermia severity, and NOA versus OA; Gali et al. [21] investigated strain elastography according to sperm-retrieval outcome; Illiano et al. [22] evaluated conventional semen parameters and abnormal-versus-normal semen phenotypes; Li et al. [23] compared NOA and OA; Rashid et al. [24] further assessed strain elastography for sperm retrieval; Rocher et al. [25] contributed the non-KS NOA-versus-OA comparison; Turna et al. [26] compared oligospermic and normospermic men; Varol et al. [27] contributed to analyses of sperm concentration, azoospermia, and testicular volume; Yavuz et al. [28] evaluated sperm concentration and azoospermia using VTIQ; and Zhang et al. [29] contributed to the NOA-versus-OA and testicular-volume analyses. For NOA analyses, non-Klinefelter syndrome (non-KS) populations were preferentially considered because KS may independently affect testicular stiffness through its distinct genetic, histological, and endocrine phenotype. Accordingly, Rocher et al. [25] contributed only the non-KS subgroup and Li et al. [23] excluded KS at enrollment, whereas Cui et al. [20] was retained as reported because a separate non-KS estimate was unavailable. The characteristics and specific quantitative contribution of each study are summarized in Table 1.

### 3.3. Risk of Bias Assessment

The revised JBI analytical cross-sectional tool was applied uniformly to all 11 included studies. The most frequent concern was incomplete control of confounding. Unit-of-analysis problems were additionally relevant where bilateral testes were analyzed as independent observations. Cui et al. had the strongest overall methodological profile, whereas Gali et al., Rocher et al., and Zhang et al. were judged at high risk of bias, mainly because of clustering/statistical-unit concerns and incomplete adjustment. Detailed assessments are presented in Table 2.

### 3.4. Semen Parameters

#### 3.4.1. Mean Stiffness Versus Sperm Concentration

**Figure 2:**
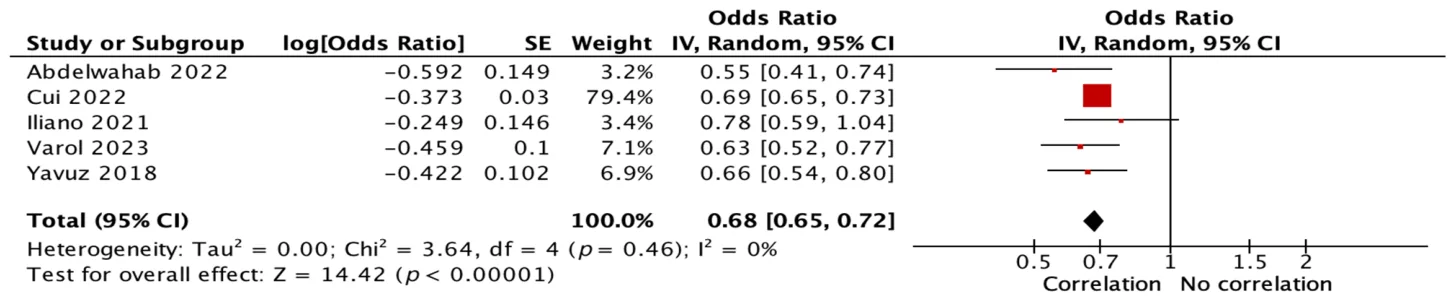
Forest plot of the correlation between mean testicular stiffness and sperm concentration. Included studies: [19,20,22,27,28]. Red squares indicate individual-study estimates, with size proportional to weight; horizontal lines indicate 95% CIs; the black diamond indicates the pooled estimate and its 95% CI.

Meta-analysis of five studies involving 1427 men showed a significant inverse association between mean testicular stiffness and sperm concentration (r = −0.37, 95% CI −0.41 to −0.32, *p* < 0.001). No statistical heterogeneity was detected (I^2^ = 0%), although the Cui cohort contributed approximately 79% of the weight (Figure 2).

#### 3.4.2. Mean Stiffness Versus Sperm Motility

**Figure 3:**
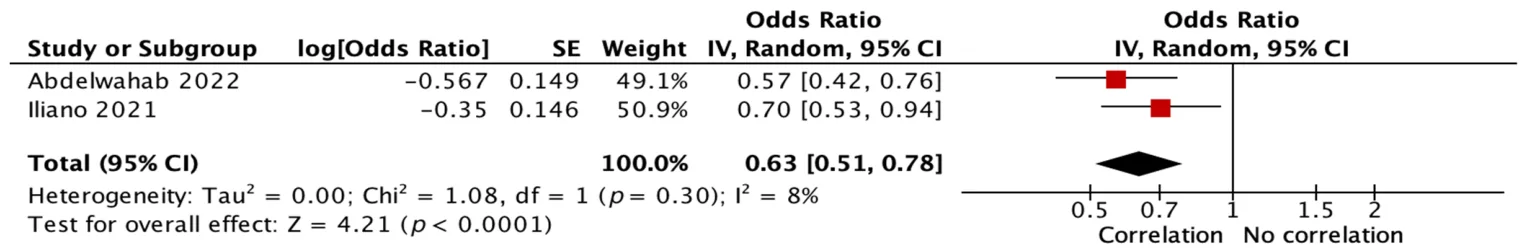
Forest plot of the correlation between mean testicular stiffness and sperm motility. Included studies: [19,22]. Red squares indicate individual-study estimates, with size proportional to weight; horizontal lines indicate 95% CIs; the black diamond indicates the pooled estimate and its 95% CI.

Meta-analysis of two studies involving 98 men showed a significant inverse association between mean testicular stiffness and sperm motility (r = −0.43, 95% CI −0.58 to −0.24, *p* < 0.001). Statistical heterogeneity was low (I^2^ = 8%) (Figure 3).

#### 3.4.3. Mean Stiffness Versus Sperm Morphology

**Figure 4:**
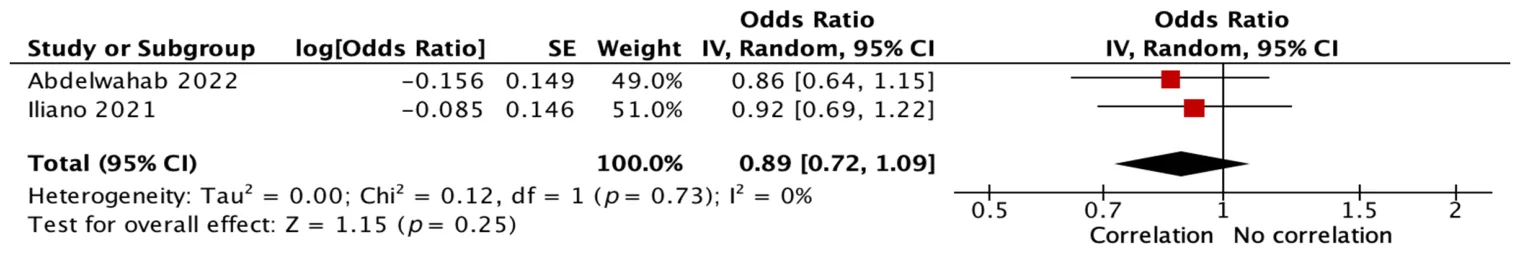
Forest plot of the correlation between mean testicular stiffness and normal sperm morphology. Included studies: [19,22]. Red squares indicate individual-study estimates, with size proportional to weight; horizontal lines indicate 95% CIs; the black diamond indicates the pooled estimate and its 95% CI.

Meta-analysis of two studies involving 98 men showed no statistically significant association between mean testicular stiffness and the percentage of sperm with normal morphology (r = −0.12, 95% CI −0.31 to 0.08, *p* = 0.25). No statistical heterogeneity was detected (I^2^ = 0%) (Figure 4).

### 3.5. Azoospermia and Semen-Phenotype Severity

#### 3.5.1. VTIQ Analysis in Azoospermia Versus Normozoospermia

**Figure 5:**
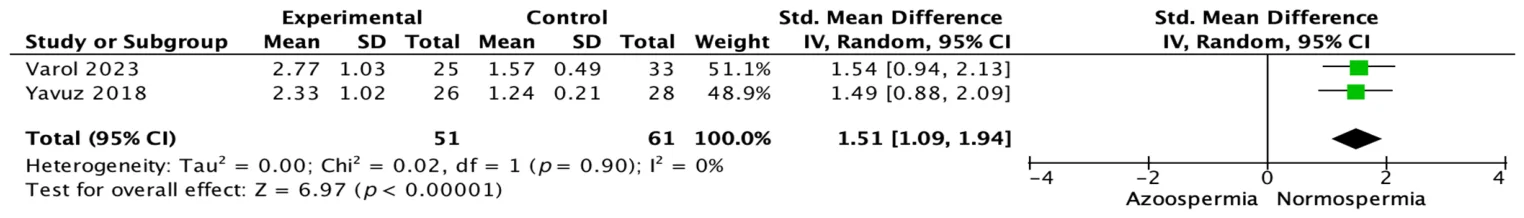
Forest plot comparing VTIQ-standardized testicular stiffness between azoospermic and normozoospermic men. This modality-specific subset also contributes to Figure 6. Included studies: [27,28]. Green squares indicate individual-study estimates, with size proportional to weight; horizontal lines indicate 95% CIs; the black diamond indicates the pooled estimate and its 95% CI.

Meta-analysis of two VTIQ studies including 51 azoospermic men and 61 normozoospermic controls showed significantly greater testicular stiffness in the azoospermic group (SMD 1.51, 95% CI 1.09–1.94, *p* < 0.001). No statistical heterogeneity was detected (I^2^ = 0%) (Figure 5).

#### 3.5.2. Standardized Stiffness in Azoospermia Versus Normozoospermia

**Figure 6:**
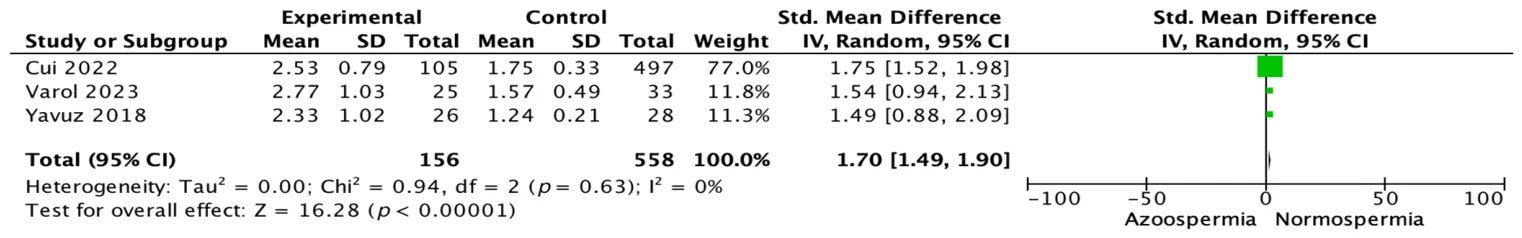
Forest plot comparing standardized testicular stiffness between azoospermic and normozoospermic men. Included studies: [20,27,28]. Green squares indicate individual-study estimates, with size proportional to weight.

Meta-analysis of three studies including 156 azoospermic men and 558 normozoospermic controls showed greater standardized testicular stiffness in azoospermia (SMD 1.70, 95% CI 1.49–1.90, *p* < 0.001). No statistical heterogeneity was detected (I^2^ = 0%) (Figure 6).

#### 3.5.3. Stiffness in Abnormal/Oligospermic Versus Normospermic

**Figure 7:**
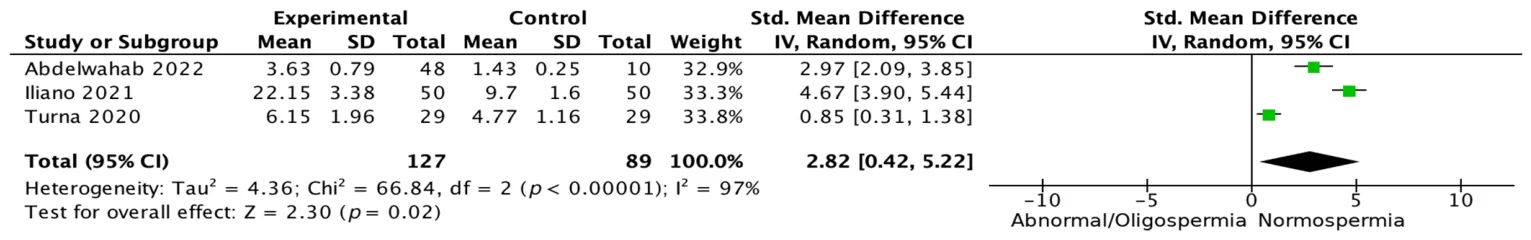
Forest plot comparing standardized testicular stiffness between abnormal/oligospermic and normospermic men. Included studies: [19,22,26]. Green squares indicate individual-study estimates, with size proportional to weight; horizontal lines indicate 95% CIs; the black diamond indicates the pooled estimate and its 95% CI.

Meta-analysis of three studies including 127 men with abnormal/oligospermic semen parameters and 89 normospermic controls showed significantly greater testicular stiffness in the abnormal-semen groups (SMD 2.817, 95% CI 0.42–5.22, *p* = 0.02). Study heterogeneity was very high (I^2^ = 97%), indicating that the magnitude of the between-group difference varied markedly across studies and should not be interpreted as a universal clinical effect size (Figure 7).

### 3.6. Stiffness in NOA Versus OA

**Figure 8:**
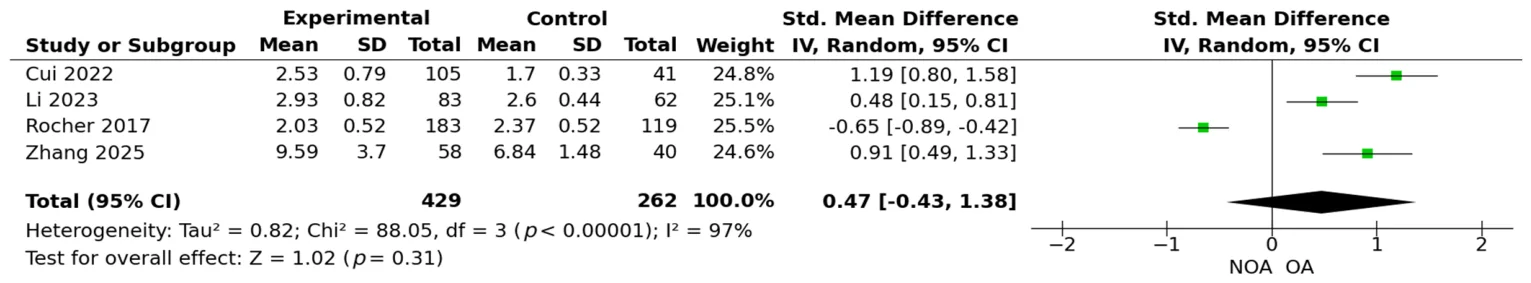
Forest plot comparing standardized testicular stiffness between non-obstructive and obstructive azoospermia. Counts combine men and testicular observations and do not represent independent patients. Included studies: [20,23,25,29]. Green squares indicate individual-study estimates, with size proportional to weight; horizontal lines indicate 95% CIs; the black diamond indicates the pooled estimate and its 95% CI.

Meta-analysis of four studies comprising 429 NOA and 262 OA analytical observations found no evidence of a difference in testicular stiffness (SMD 0.47, 95% CI −0.43 to 1.38, *p* = 0.31). The estimate was highly imprecise, and heterogeneity was very high (I^2^ = 97%) (Figure 8). These data do not establish equivalence and cannot rule out a clinically important difference. The totals combine 188 NOA and 103 OA men from Cui et al. and Li et al. with 241 NOA and 159 OA testicular observations from Rocher et al. and Zhang et al.; the synthesis should therefore be interpreted as exploratory. Rocher et al. contributed only the non-Klinefelter NOA subgroup; where a clean non-KS subgroup was not separately available, this limitation was retained explicitly rather than assuming that all NOA observations were etiologically equivalent.

### 3.7. Sperm Retrieval and Testicular Volume

#### 3.7.1. Ancillary Strain-Ratio Retrieval Analysis

**Figure 9:**
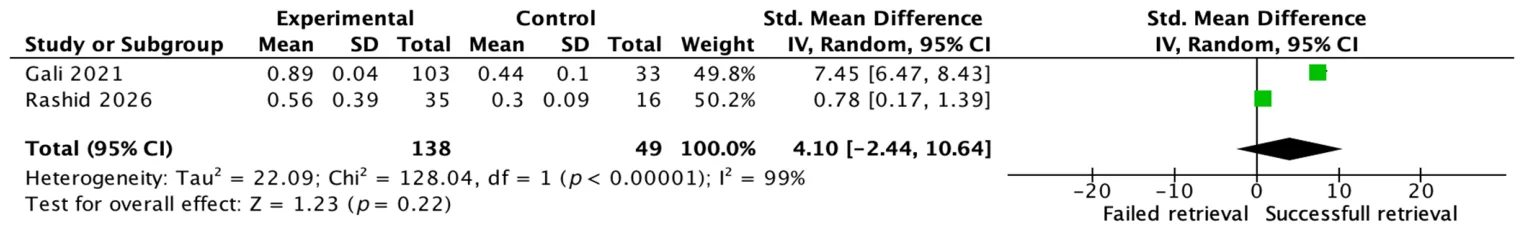
Forest plot comparing strain ratio according to sperm-retrieval outcome in azoospermia. Gali et al. contributed testes and Rashid et al. contributed men; the totals are analytical observations. The pooled estimate is highly imprecise and does not establish equivalence. Included studies: [21,24]. Green squares indicate individual-study estimates, with size proportional to weight; horizontal lines indicate 95% CIs; the black diamond indicates the pooled estimate and its 95% CI.

Meta-analysis of two strain-elastography studies including 138 failed and 49 successful sperm-retrieval analytical observations found no evidence of a difference in strain ratio (SMD 4.10, 95% CI −2.44 to 10.64, *p* = 0.22). These observations comprise 136 biopsied testes in Gali et al. and 51 men in Rashid et al. Heterogeneity was very high (I^2^ = 99%) (Figure 9), and the estimate was highly imprecise. The wide confidence interval does not demonstrate equivalence and cannot rule out clinically important differences in either direction. Because both studies used strain elastography rather than conventional SWE, this analysis was considered ancillary and was not interpreted as direct evidence of SWE-specific prediction.

#### 3.7.2. Testicular Stiffness Versus Testicular Volume

**Figure 10:**
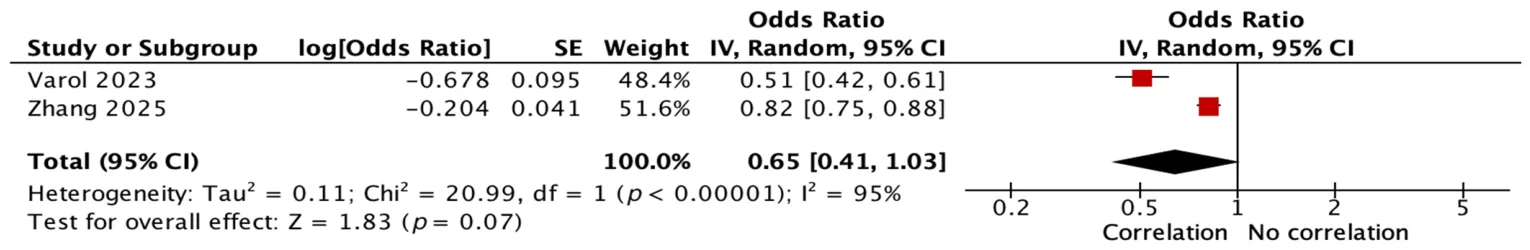
Forest plot of the correlation between testicular stiffness and testicular volume. Included studies: [27,29]. Red squares indicate individual-study estimates, with size proportional to weight; horizontal lines indicate 95% CIs; the black diamond indicates the pooled estimate and its 95% CI.

Meta-analysis of two studies comprising 113 infertile men in Varol et al. and 608 testicular observations in Zhang et al. yielded an imprecise inverse estimate of the relationship between testicular stiffness and testicular volume (r = −0.41, 95% CI −0.72 to 0.03, *p* = 0.07). Heterogeneity was very high (I^2^ = 95%) (Figure 10), and the mixed patient-level and testis-level statistical units further limit interpretation. This synthesis is exploratory and cannot establish a common magnitude of association or support clinical recommendations.

## 4. Discussion

### 4.1. Principal Findings

The data do not support a single, uniform account of testicular stiffness. The sperm-concentration analysis provides the largest evidence base: five studies involving 1427 men produced an inverse correlation (r = −0.37, 95% CI −0.41 to −0.32), with no statistical heterogeneity detected. However, approximately 79% of the weight came from Cui et al., limiting independent replication. The narrow pooled confidence interval is driven largely by this cohort and should not be taken as evidence of reproducibility across independent populations. Motility pointed in the same direction, although that estimate rested on only two studies. No association was detected for morphology. These differences support analyzing semen endpoints separately, particularly because morphological classification remains sensitive to biological and laboratory variation [5,30].

The comparisons between clinical groups were less tidy. Higher stiffness was observed in azoospermic than in normozoospermic men, and abnormal/oligospermic groups also had higher stiffness, but the magnitude of the latter contrast varied sharply (I^2^ = 97%). The NOA–OA estimate was highly imprecise and does not establish similar stiffness in the two groups. Both this comparison and the stiffness–volume association remain exploratory because of their marked heterogeneity and mixed statistical units. SWE appears to detect part of the spermatogenic phenotype; the present data do not show that this signal corresponds to one disease process or one transferable threshold.

### 4.2. Semen Phenotype and Testicular Mechanics

A simple fibrosis explanation would be attractive, but the available evidence does not justify it. Elastography samples the mechanical behavior of the seminiferous and interstitial compartments together. Oxidative injury may affect both germ cells and their tissue environment [31,32]. Recent mechanistic reviews reinforce the relevance of redox imbalance to sperm dysfunction [33]. Environmental toxicants have also been associated with altered semen parameters [34,35], although these observations do not establish an elastographic mechanism. Tubular atrophy and interstitial remodeling are also plausible contributors. None of these mechanisms is measured directly by SWE. The elastogram remains a macroscopic signal rather than a virtual biopsy [36], and the value recorded at the console reflects acquisition physics as well as tissue biology [37,38].

That distinction helps to interpret the I^2^ of 97% in the abnormal-versus-normal semen analysis. The studies agreed on the direction of the difference, but not on its scale. Fibrosis, vascular loading, edema, cellular loss, and operator-dependent acquisition need not affect stiffness in the same way [39]. Hypoxia adds another influence in varicocele [40]. An average difference between groups can therefore be real even when a numerical cutoff is not portable from one platform to another.

The null result for morphology is not merely an inconvenient exception. Concentration approximates sperm output, whereas motility also incorporates post-testicular function. Morphology is more dependent on observer and laboratory practice. Treating these endpoints as interchangeable components of “semen quality” would erase precisely the distinction that emerged in the pooled data [5,7,30].

### 4.3. Clinical Interpretation in Varicocele and Azoospermia

Varicocele makes the difference between tissue change and diagnostic specificity particularly visible. Venous reflux alters the local thermal, oxygen, and redox environment, yet varicocele-associated infertility is not explained by venous dilatation alone [41]. Oxidative injury, disturbed homeostasis, sperm DNA damage, and impaired spermatogenesis are recurrent elements of the proposed biology [42,43,44,45,46]. Wang et al. showed that varicocele-bearing testes differ mechanically from contralateral and normal testes [14]. Our analysis asked a broader question: whether stiffness follows semen impairment among men evaluated for infertility, including those without varicocele.

This difference in scope matters after treatment. Repair can improve conventional semen parameters in selected men [47], and later syntheses report reductions in sperm DNA fragmentation with possible benefits for assisted reproduction [48,49]. A change in stiffness would be interesting only if it accompanied one of those outcomes. Otherwise, it would describe image behavior rather than recovery of reproductive function.

The azoospermia analyses leave the diagnostic specificity of elastography uncertain. Stiffness differed between azoospermic and normozoospermic men, an extreme phenotypic contrast, whereas the highly heterogeneous NOA–OA synthesis found no evidence of a difference and was too imprecise to establish equivalence. Variation in stiffness across etiologies is biologically credible. Intratesticular sperm production is generally preserved in OA, whereas NOA encompasses several histological and endocrine patterns [50] and an expanding range of genetic disorders [51]. A recent review of exome sequencing further illustrates the genetic diversity of quantitative and qualitative spermatogenic defects [52]. These different forms of failure need not share the same mechanical phenotype. The imprecise pooled comparison neither excludes clinically important NOA–OA differences nor establishes the diagnostic utility of SWE for this distinction. Etiology must still be established through clinical assessment and appropriately directed endocrine, genetic, and conventional imaging investigations [2,3,50,51]. The wider NOA literature reinforces this point: pooled retrieval rates are close to one half, yet routine variables remain inaccurate for an individual patient [53], and evidence for hormonal pretreatment is heterogeneous [54]. A cohort-specific stiffness cutoff would not resolve that uncertainty.

### 4.4. Sperm Retrieval and Clinical Integration

The retrieval analysis is especially vulnerable to overinterpretation. Only two studies contributed, both measuring strain ratio rather than conventional SWE [21,24]. Failed retrieval was associated with higher values in each study, but the pooled SMD of 4.10 had a very wide 95% CI (−2.44 to 10.64), with I^2^ = 99%. These data do not establish equivalence and cannot rule out clinically important differences in either direction. They also do not establish prediction of micro-TESE or pTESA. Retrieval in NOA is inherently difficult to forecast [55]. Chromosomal etiology modifies the probability [56], and spermatogenesis may be focal, which is precisely why microdissection remains useful [57].

Several recent models have tried to combine weak predictors. A multicenter nomogram of more than 3000 men included age and testicular volume [58]; machine-learning studies have added complexity without consistently adding external validation [59]. The appropriate benchmark for elastography is not whether stiffness is associated with retrieval in the development cohort. It is whether stiffness improves prediction beyond routine data in a separate cohort. Individualized retrieval strategies remain necessary in selected settings [60], endocrine markers alone are insufficient [61], and the role of varicocele repair before retrieval in NOA remains unsettled [62,63]. No imaging measurement currently carries enough information to decide whether surgery should be offered.

Testicular volume helps to place stiffness in context, but it does not duplicate it. Their pooled association was imprecise and highly heterogeneous (I^2^ = 95%), providing only exploratory evidence that cannot establish a relationship transferable across clinical populations. A small testis may reflect longstanding loss of spermatogenic tissue; congestion can change mechanical behavior before volume falls. Similar limitations are familiar in NOA, where volume contributes to prognosis without determining retrieval for an individual man [53,55,58]. In practice, elastography would enter an examination that already includes B-mode structure and volume, with Doppler used when vascular information is relevant [11,12,13]. Its value cannot be judged by fitting a larger collection of imaging variables to the same cohort. The useful question is whether the stiffness measurement changes the interpretation reached from the conventional examination.

### 4.5. Standardization and Research Priorities

Technical heterogeneity is not background noise in this literature; it changes what the number means. The EFSUMB scrotal ultrasound guideline and foundational WFUMB and EFSUMB elastography recommendations provide the framework for standardized acquisition and terminology [12,37,38,39]. Stiffness varies across platforms and with the way the operator samples the testis. Dedicated testicular work confirms that acquisition strategy alone can materially alter the result [64]. Reports rarely need an exhaustive technical inventory, but they must provide enough information to repeat the measurement, particularly the placement of the region of interest and the summary of repeated acquisitions.

The statistical unit creates a different distortion. Two testes from the same man are correlated. Counting them as independent observations narrows confidence intervals artificially unless clustering is addressed or a patient-level summary is specified.

Use of one revised JBI tool made the appraisal consistent [18], but it also exposed limited adjustment for confounding. Underlying diagnosis and endocrine or exposure-related factors may influence both stiffness and reproductive phenotype [6,51,65]. Better phenotyping cannot be reconstructed reliably after the study has ended. It needs to shape recruitment and data collection from the outset, particularly in multicenter work.

Further work should follow the unanswered biological questions rather than reproduce another cross-sectional cutoff. Sperm DNA fragmentation (SDF) is one such question. It is assay-dependent and recommended for selective use [66,67,68,69,70], and it reflects damage within the gamete rather than the mechanical state of the testis [71]. Measuring SDF and stiffness prospectively in the same men would show whether the imaging signal contains information not already present in semen testing.

Pregnancy and live birth are more meaningful but less easily attributed to a male imaging finding because the female partner and subsequent treatment also determine the outcome. Varicocele studies have begun to report these endpoints [49], and outcome estimates are available after ICSI with surgically retrieved sperm [53]. Elastography studies have rarely followed patients that far. Until they do, stiffness is best described as part of testicular phenotyping rather than as a measure of fertility.

Two study designs would move the field forward for different reasons. In varicocele, repeated acquisitions before and after treatment could show whether mechanical change accompanies a clinical response, with careful patient selection as emphasized in guidance on antioxidant therapy [72]. In NOA, elastographic maps could be matched spatially with micro-TESE sampling sites and histology. The first design tests change over time; the second tests whether the apparent mechanical signal has a focal biological counterpart.

### 4.6. Strengths and Limitations

The review was kept close to the quantitative evidence. Every included study carried non-zero weight in at least one synthesis, and the RevMan dataset is reproducible. Semen endpoints were analyzed separately, correlations were pooled on the Fisher-z scale, and strain elastography was not merged with SWE. Risk of bias was assessed with the same JBI tool throughout [15,18].

The evidence base remains small. Most outcomes drew on two to five studies, and Cui et al. contributed approximately 79% of the sperm-concentration weight, limiting independent replication of that association. Heterogeneity is imprecisely estimated with few studies, and normal-approximation confidence intervals may understate uncertainty [16,73]. Recruitment and acquisition protocols varied, and potential confounders were incompletely reported or controlled. Some secondary syntheses combined patient-level and testis-level summaries without a reconstructable adjustment for paired-testis dependence. Their nominal confidence intervals may be too narrow, and the contribution of testis-level studies may be overstated; random-effects pooling does not resolve this problem. Several inputs also had to be derived from published summary statistics. These concerns are less pronounced for the patient-level semen correlations and most consequential for the abnormal-semen, NOA-versus-OA, retrieval, and stiffness–volume estimates, all of which showed very high heterogeneity. These highly heterogeneous syntheses are exploratory and cannot support clinical recommendations or estimates of a transferable clinical effect. Narrative studies could provide context but, by design, did not enter the pooled evidence base.

Male infertility has broader health and psychosocial associations [74,75], but those associations should not be projected onto an elastographic measurement. The claim supported here is narrower. Stiffness reflects part of the reproductive phenotype; whether it changes a clinical assessment after the standard work-up remains unanswered.

## 5. Conclusions

Available evidence suggests inverse associations of testicular stiffness with sperm concentration and motility and higher stiffness in azoospermic than normozoospermic men. Few studies per outcome, the dominance of Cui et al. in the concentration analysis, and technical and statistical heterogeneity limit confidence in the pooled estimates. The NOA–OA, sperm-retrieval, and stiffness–volume analyses are exploratory. Their imprecision, high heterogeneity, and unresolved paired-testis dependence preclude clinical recommendations based on these pooled estimates. These findings support further evaluation of elastography as an adjunct to testicular assessment. They do not establish diagnostic thresholds, reliable differentiation of NOA from OA, or the ability to guide sperm-retrieval decisions for individual patients. Standardized acquisition, patient-level analysis, and external validation remain necessary.

## Figures and Tables

**Table 1:** Characteristics of studies evaluating testicular elastography in male infertility.

Study	Design	Population/Groups Used	Elastography	Final Comments
Abdelwahab 2022 [19]	Case-control	48 unilateral-varicocele men with abnormal semen plus 10 normospermic volunteers	Contralateral SWE, kPa	Greater testicular stiffness was associated with lower sperm concentration and motility, while men with abnormal semen parameters showed substantially greater stiffness than normospermic controls; the association with morphology was weak.
Cui 2022 [20]	Single-center cross-sectional	1116 men in IVF/infertility pathway; NOA n = 105, OA n = 41	Emean/Emax, kPa	The largest included study showed an inverse association between stiffness and sperm concentration and markedly greater stiffness in azoospermic than normozoospermic men; its NOA–OA comparison contributed to the substantial heterogeneity of that analysis.
Gali 2021 [21]	Cross-sectional	98 azoospermic men/196 testes; 136 biopsied testes; final contrast 103 failed vs. 33 successful	Strain ratio	Failed retrieval was associated with a higher strain ratio than successful retrieval in testis-level observations; these data contributed only to the separate ancillary strain-ratio synthesis.
Illiano 2021 [22]	Prospective case-control	50 OAT men and 50 normozoospermic controls	Mean SWE, kPa	OAT was characterized by substantially greater testicular stiffness, with inverse relationships with sperm concentration and motility; no statistically significant association with normal morphology was detected.
Li 2023 [23]	Observational diagnostic	145 azoospermic infertile men: NOA n = 83, OA n = 62	SWE, kPa	NOA showed greater testicular stiffness than OA, supporting a potential relationship between parenchymal stiffness and impaired spermatogenesis, although the pooled NOA–OA effect was ultimately inconsistent across studies.
Rashid 2026 [24]	Observational diagnostic	51 azoospermic men: 35 negative vs. 16 positive pTESA	Strain ratio	Higher strain ratios were observed in men with unsuccessful sperm retrieval, corroborating the direction of the Gali study but with substantial between-study heterogeneity.
Rocher 2017 [25]	Prospective; larger cohort of normal and infertile men	Non-KS NOA n = 183 testes and OA n = 119 testes from the published subgroups	2D-SWE, kPa	In contrast to several other cohorts, non-KS NOA showed lower stiffness than OA, contributing importantly to the very high heterogeneity of the pooled NOA–OA comparison.
Turna 2020 [26]	Observational comparative	58 men with left varicocele plus 29 healthy controls; meta-analysis: 29 oligospermic vs. 29 normospermic	SWE, kPa	Oligospermic men showed greater testicular stiffness than normospermic men, supporting the overall association between abnormal semen quality and increased stiffness.
Varol 2023 [27]	Prospective observational	113 infertile men; azo n = 25, normo n = 33 in subgroup analysis	ARFI/VTIQ, m/s	Higher SWV was associated with poorer sperm concentration and azoospermia, while stiffness was inversely related to testicular volume, providing evidence across both functional and structural endpoints.
Yavuz 2018 [28]	Prospective	100 men; 90 infertility referrals	VTIQ/SWV, m/s	Higher SWV accompanied more severe impairment of spermatogenesis, contributing to both the inverse sperm-concentration association and the greater stiffness observed in azoospermia.
Zhang 2025 [29]	Prospective pilot/cross-sectional	360 participants; NOA 58 and OA 40 testes; volume correlation 608 testes	SWE-derived elasticity, kPa	NOA showed greater stiffness than OA and stiffness was inversely related to testicular volume; however, testis-level clustering and marked heterogeneity limit the precision of both pooled effects.

Abbreviations: 2D-SWE, two-dimensional shear-wave elastography; ARFI, acoustic radiation force impulse; Emax, maximum elastic modulus; Emean, mean elastic modulus; IVF, in vitro fertilization; kPa, kilopascal; KS, Klinefelter syndrome; m/s, meters per second; NOA, non-obstructive azoospermia; OA, obstructive azoospermia; OAT, oligoasthenoteratozoospermia; pTESA, percutaneous testicular sperm aspiration; SWD, shear-wave dispersion; SWE, shear-wave elastography; SWV, shear-wave velocity; VTIQ, Virtual Touch Tissue Imaging Quantification. Counts refer to men unless specifically identified as testes. In Zhang et al., SWE-derived elasticity in kPa was synthesized; SWD-derived viscosity and dispersion slope were not pooled.

**Table 2:** Risk-of-bias assessment using the revised JBI analytical cross-sectional tool.

**Study**	**Q1**	**Q2**	**Q3**	**Q4**	**Q5**	**Q6**	**Q7**	**Q8**	**Overall**
Abdelwahab 2022 [19]	Y	Y	Y	Y	U	N	Y	Y	Moderate
Cui 2022 [20]	Y	Y	Y	Y	Y	Y	Y	Y	Low
Gali 2021 [21]	Y	Y	U	Y	U	N	N	Y	High
Illiano 2021 [22]	Y	Y	Y	Y	Y	N	Y	Y	Moderate
Li 2023 [23]	Y	Y	Y	Y	Y	N	Y	Y	Moderate
Rashid 2026 [24]	Y	Y	U	Y	Y	N	Y	Y	Moderate
Rocher 2017 [25]	Y	Y	Y	Y	Y	N	N	Y	High
Turna 2020 [26]	Y	Y	Y	Y	U	N	Y	Y	Moderate
Varol 2023 [27]	Y	Y	Y	Y	U	N	Y	Y	Moderate
Yavuz 2018 [28]	Y	Y	Y	Y	Y	N	Y	Y	Moderate
Zhang 2025 [29]	Y	Y	Y	Y	Y	Y	N	Y	High

Notes: Q1, inclusion criteria; Q2, objective condition measurement; Q3, valid/reliable exposure measurement; Q4, valid/reliable outcome measurement; Q5, confounders identified; Q6, confounding strategy; Q7, statistical analysis; Q8, participants/setting described; Y, yes; N, no; U, unclear.

## Data Availability

All data analyzed in this study were extracted from the published reports cited in the article. The analytical dataset is available from the corresponding author upon reasonable request.

## Abbreviations

Abbreviation	Definition
2D-SWE	Two-dimensional shear-wave elastography
AMH	Anti-Müllerian hormone
ARFI	Acoustic radiation force impulse
ART	Assisted reproductive technology
AUC	Area under the curve
CI	Confidence interval
EFSUMB	European Federation of Societies for Ultrasound in Medicine and Biology
Emax	Maximum elastic modulus
Emean	Mean elastic modulus
FSH	Follicle-stimulating hormone
ICSI	Intracytoplasmic sperm injection
IVF	In vitro fertilization
JBI	Joanna Briggs Institute
KS	Klinefelter syndrome
MD	Mean difference
micro-TESE	Microdissection testicular sperm extraction
NOA	Non-obstructive azoospermia
OA	Obstructive azoospermia
OAT	Oligoasthenoteratozoospermia
PICOS	Population, intervention/index test, comparison, outcomes, and study type
PRISMA	Preferred Reporting Items for Systematic Reviews and Meta-Analyses
pTESA	Percutaneous testicular sperm aspiration
ROI	Region of interest
SDF	Sperm DNA fragmentation
SMD	Standardized mean difference
SWD	Shear-wave dispersion
SWE	Shear-wave elastography
SWV	Shear-wave velocity
VTIQ	Virtual Touch Tissue Imaging Quantification
WFUMB	World Federation for Ultrasound in Medicine and Biology
WHO	World Health Organization

## References

[1] Eisenberg M.L., Esteves S.C., Lamb D.J., Hotaling J.M., Giwercman A., Hwang K., Cheng Y.-S. (2023). Male infertility. Nat. Rev. Dis. Primers.

[2] Minhas S., Boeri L., Capogrosso P., Cocci A., Corona G., Dinkelman-Smit M., Falcone M., Jensen C.F., Gül M., Kalkanli A. (2025). European Association of Urology Guidelines on Male Sexual and Reproductive Health: 2025 Update on Male Infertility. Eur. Urol..

[3] Brannigan R.E., Hermanson L., Kaczmarek J., Kim S.K., Kirkby E., Tanrikut C. (2024). Updates to Male Infertility: AUA/ASRM Guideline. J. Urol..

[4] World Health Organization (2025). Guideline for the Prevention, Diagnosis and Treatment of Infertility.

[5] World Health Organization (2021). WHO Laboratory Manual for the Examination and Processing of Human Semen.

[6] Wyrwoll M.J., van der Heijden G.W., Krausz C., Aston K.I., Kliesch S., McLachlan R., Ramos L., Conrad D.F., O’Bryan M.K., Veltman J.A. (2024). Improved phenotypic classification of male infertility to promote discovery of genetic causes. Nat. Rev. Urol..

[7] Björndahl L., Kirkman Brown J., other Editorial Board Members of the WHO Laboratory Manual for the Examination and Processing of Human Semen (2022). The sixth edition of the WHO Laboratory Manual for the Examination and Processing of Human Semen: Ensuring quality and standardization in basic examination of human ejaculates. Fertil. Steril..

[8] Levine H., Jørgensen N., Martino-Andrade A., Mendiola J., Weksler-Derri D., Jolles M., Pinotti R., Swan S.H. (2023). Temporal trends in sperm count: A systematic review and meta-regression analysis of samples collected globally in the 20th and 21st centuries. Hum. Reprod. Update.

[9] Agarwal A., Baskaran S., Parekh N., Cho C.-L., Henkel R., Vij S., Arafa M., Selvam M.K.P., Shah R. (2021). Male infertility. Lancet.

[10] Sang Q., Ray P.F., Wang L. (2023). Understanding the genetics of human infertility. Science.

[11] Lotti F., Studniarek M., Balasa C., Belfield J., De Visschere P., Freeman S., Kozak O., Markiet K., Ramanathan S., Richenberg J. (2025). The role of the radiologist in the evaluation of male infertility: Recommendations of the ESUR-SPIWG for scrotal imaging. Eur. Radiol..

[12] Bertolotto M., Freeman S., Huang D.Y., Sidhu P.S., Campo I., Clevert D.-A., Dogra V., Drudi F.M., Fischer T., Isidori A.M. (2026). EFSUMB Guidelines on Multiparametric Ultrasound of the Scrotum. Ultraschall Med..

[13] Cantisani V., Di Leo N., Bertolotto M., Fresilli D., Granata A., Polti G., Polito E., Pacini P., Guiban O., Del Gaudio G. (2021). Role of multiparametric ultrasound in testicular focal lesions and diffuse pathology evaluation, with particular regard to elastography: Review of literature. Andrology.

[14] Wang L., Huang X., Ding Y. (2025). Shear wave elastography in assessing stiffness and volume of varicocele-affected and normal testes: A systematic review and meta-analysis. Basic Clin. Androl..

[15] Page M.J., McKenzie J.E., Bossuyt P.M., Boutron I., Hoffmann T.C., Mulrow C.D., Shamseer L., Tetzlaff J.M., Akl E.A., Brennan S.E. (2021). The PRISMA 2020 statement: An updated guideline for reporting systematic reviews. BMJ.

[16] Deeks J.J., Higgins J.P., Altman D.G., McKenzie J.E., Veroniki A.A., Higgins J.P.T., Thomas J., Chandler J., Cumpston M., Li T., Page M.J., Welch V.A. (2024). Chapter 10: Analysing data and undertaking meta-analyses. Cochrane Handbook for Systematic Reviews of Interventions, Version 6.5.

[17] Hozo S.P., Djulbegovic B., Hozo I. (2005). Estimating the mean and variance from the median, range, and the size of a sample. BMC Med. Res. Methodol..

[18] Barker T.H., Hasanoff S., Aromataris E., Stone J.C., Leonardi-Bee J., Sears K., Klugar M., Tufanaru C., Moola S., Liu X.-L. (2026). The revised JBI critical appraisal tool for the assessment of risk of bias for analytical cross-sectional studies. JBI Evid. Synth..

[19] Abdelwahab K.M., Desoky E.A., Abu Bakr M., El-Samed K.A., Rashed M.M.M.E., Elnagar A.E., Ibrahem M.S.M. (2022). Shear Wave Elastography of the Contralateral Apparently Normal Testis in Patients with Unilateral Varicocele and Abnormal Semen Parameters. Egypt. J. Hosp. Med..

[20] Cui J., Du Q., Fu W. (2022). Application of real-time shear wave elastography in the assessment of male infertility. Quant. Imaging Med. Surg..

[21] Gali S., Alkhursan M.E., Mahdi M.A., Al Saad R.H. (2021). Role of sonoelastography in predicting positive sperm retrieving in patients with non-obstructive azoospermia. Indian J. Forensic Med. Toxicol..

[22] Illiano E., Trama F., Ruffo A., Romeo G., Riccardo F., Crocetto F., Iacono F., Costantini E. (2021). Testicular shear wave elastography in oligo-astheno-teratozoospermic individuals: A prospective case-control study. Int. Urol. Nephrol..

[23] Li X., Tian R.H., Li P., Li C.-X., Yao M.-H., Yao C.-C., Wang X.-B., Jiang L.-R., Li Z., Wu R. (2023). Ultrasonographic evaluation of the rete testis thickness: A promising approach to differentiate obstructive from nonobstructive azoospermia. Asian J. Androl..

[24] Rashid A.O., Faraj F.M., Ahmed S.M., Othman D.S., Ali A.M.M., Omer A.A., Salahadin A.A., Al Bayani A.S.S. (2026). Predictive value of testicular elastography on the outcome of percutaneous testicular sperm aspiration in patients with azoospermia. Sci. Rep..

[25] Rocher L., Criton A., Gennisson J.L., Izard V., Ferlicot S., Tanter M., Benoit G., Bellin M.F., Correas J.-M. (2017). Testicular shear wave elastography in normal and infertile men: A prospective study on 601 patients. Ultrasound Med. Biol..

[26] Turna O., Aybar M.D. (2020). Testicular stiffness in varicocele: Evaluation with shear wave elastography. Ultrasonography.

[27] Varol C., Çolakoğlu Er H., Demir M. (2023). The relationship between testis ARFI elastography values and sperm count in infertile patients. Harran Univ. Med. Fac. J..

[28] Yavuz A., Yokuş A., Taken K., Batur A., Özgökçe M., Arslan H. (2018). Reliability of testicular stiffness quantification using shear wave elastography in predicting male fertility: A preliminary prospective study. Med. Ultrason..

[29] Zhang S., Wu M., Xu L., Wang H., Jiang L. (2025). High-frequency ultrasound shear wave dispersion imaging for male infertility: A pilot study. Quant. Imaging Med. Surg..

[30] Barratt C.L.R., Björndahl L., De Jonge C.J., Lamb D.J., Osorio Martini F., McLachlan R., Oates R.D., Van Der Poel S., St John B., Sigman M. (2017). The diagnosis of male infertility: An analysis of the evidence to support the development of global WHO guidance—Challenges and future research opportunities. Hum. Reprod. Update.

[31] Evans E.P.P., Scholten J.T.M., Mzyk A., Reyes-San-Martin C., Llumbet A.E., Hamoh T., Arts E.G., Schirhagl R., Cantineau A.E. (2021). Male subfertility and oxidative stress. Redox Biol..

[32] Barati E., Nikzad H., Karimian M. (2020). Oxidative stress and male infertility: Current knowledge of pathophysiology and role of antioxidant therapy in disease management. Cell Mol. Life Sci..

[33] Wang Y., Fu X., Li H. (2025). Mechanisms of oxidative stress-induced sperm dysfunction. Front. Endocrinol..

[34] Giulioni C., Maurizi V., Scarcella S., Di Biase M., Iacovelli V., Galosi A.B., Castellani D. (2021). Do environmental and occupational exposure to pyrethroids and organophosphates affect human semen parameters? Results of a systematic review and meta-analysis. Andrologia.

[35] Giulioni C., Maurizi V., De Stefano V., Polisini G., Teoh J.Y.-C., Milanese G., Galosi A.B., Castellani D. (2023). The influence of lead exposure on male semen parameters: A systematic review and meta-analysis. Reprod. Toxicol..

[36] Sigrist R.M.S., Liau J., Kaffas A.E., Chammas M.C., Willmann J.K. (2017). Ultrasound elastography: Review of techniques and clinical applications. Theranostics.

[37] Bamber J., Cosgrove D., Dietrich C.F., Fromageau J., Bojunga J., Calliada F., Cantisani V., Correas J.-M., D’Onofrio M., Drakonaki E.E. (2013). EFSUMB guidelines and recommendations on the clinical use of ultrasound elastography. Part 1: Basic principles and technology. Ultraschall Med..

[38] Shiina T., Nightingale K.R., Palmeri M.L., Hall T.J., Bamber J.C., Barr R.G., Castera L., Choi B.I., Chou Y.-H., Cosgrove D. (2015). WFUMB Guidelines and Recommendations for Clinical Use of Ultrasound Elastography: Part 1: Basic Principles and Terminology. Ultrasound Med. Biol..

[39] Cosgrove D., Piscaglia F., Bamber J., Bojunga J., Correas J.-M., Gilja O.H., Klauser A.S., Sporea I., Calliada F., Cantisani V. (2013). EFSUMB guidelines and recommendations on the clinical use of ultrasound elastography. Part 2: Clinical applications. Ultraschall Med..

[40] Lord T. (2025). Pathophysiological effects of hypoxia on testis function and spermatogenesis. Nat. Rev. Urol..

[41] Jensen C.F.S., Østergren P., Dupree J.M., Ohl D.A., Sønksen J., Fode M. (2017). Varicocele and male infertility. Nat. Rev. Urol..

[42] Agarwal A., Hamada A., Esteves S.C. (2012). Insight into oxidative stress in varicocele-associated male infertility: Part 1. Nat. Rev. Urol..

[43] Hamada A., Esteves S.C., Agarwal A. (2013). Insight into oxidative stress in varicocele-associated male infertility: Part 2. Nat. Rev. Urol..

[44] Hassanin A.M., Ahmed H.H., Kaddah A.N. (2018). A global view of the pathophysiology of varicocele. Andrology.

[45] Arya D., Balasinor N., Singh D. (2022). Varicocoele-associated male infertility: Cellular and molecular perspectives of pathophysiology. Andrology.

[46] Cho C.L., Esteves S.C., Agarwal A. (2016). Novel insights into the pathophysiology of varicocele and its association with reactive oxygen species and sperm DNA fragmentation. Asian J. Androl..

[47] Agarwal A., Cannarella R., Saleh R., Boitrelle F., Gül M., Toprak T., Salvio G., Arafa M., Russo G.I., Harraz A.M. (2023). Impact of varicocele repair on semen parameters in infertile men: A systematic review and meta-analysis. World J. Mens. Health.

[48] Cannarella R., Shah R., Ko E., Kavoussi P., Rambhatla A., Hamoda T.A.-A.A.-M., Saleh R., Harraz A.M., Calogero A.E., Durairajanayagam D. (2025). Effects of Varicocele Repair on Testicular Endocrine Function: A Systematic Review and Meta-Analysis. World J. Mens. Health.

[49] Shah R., Agarwal A., Kavoussi P., Rambhatla A., Saleh R., Cannarella R., Harraz A.M., Boitrelle F., Kuroda S., Hamoda T.A.-A.A.-M. (2023). Consensus and Diversity in the Management of Varicocele for Male Infertility: Results of a Global Practice Survey and Comparison with Guidelines and Recommendations. World J. Mens. Health.

[50] Andrade D.L., Viana M.C., Esteves S.C. (2021). Differential diagnosis of azoospermia in men with infertility. J. Clin. Med..

[51] Houston B.J., Riera-Escamilla A., Wyrwoll M.J., Salas-Huetos A., Xavier M.J., Nagirnaja L., Friedrich C., Conrad D.F., I Aston K., Krausz C. (2022). A systematic review of the validated monogenic causes of human male infertility: 2020 update and a discussion of emerging gene-disease relationships. Hum. Reprod. Update.

[52] Riera-Escamilla A., Nagirnaja L. (2025). Utility of exome sequencing in primary spermatogenic disorders: From research to diagnostics. Andrology.

[53] Corona G., Minhas S., Giwercman A., Bettocchi C., Dinkelman-Smit M., Dohle G., Fusco F., Kadioglou A., Kliesch S., Kopa Z. (2019). Sperm recovery and ICSI outcomes in men with non-obstructive azoospermia: A systematic review and meta-analysis. Hum. Reprod. Update.

[54] Tharakan T., Corona G., Foran D., Salonia A., Sofikitis N., Giwercman A., Krausz C., Yap T., Jayasena C.N., Minhas S. (2022). Does hormonal therapy improve sperm retrieval rates in men with non-obstructive azoospermia: A systematic review and meta-analysis. Hum. Reprod. Update.

[55] Arshad M.A., Majzoub A., Esteves S.C. (2020). Predictors of surgical sperm retrieval in non-obstructive azoospermia: Summary of current literature. Int. Urol. Nephrol..

[56] Majzoub A., Arafa M., Clemens H., Imperial J., Leisegang K., Khalafalla K., Agarwal A., Henkel R., Elbardisi H. (2022). A systematic review and meta-analysis exploring the predictors of sperm retrieval in patients with non-obstructive azoospermia and chromosomal abnormalities. Andrologia.

[57] Dabaja A.A., Schlegel P.N. (2013). Microdissection testicular sperm extraction: An update. Asian J. Androl..

[58] Ceyhan E., Kayra M.V., Ates E.G., Kizilkan Y., Altan M., Yildirim O., Gultekin M.H., Akdogan N., Hasirci E., Cicek T. (2025). A Nomogram Predicting Testicular Sperm Extraction Success in Men With Non-obstructive Azoospermia: A Multi-center Study. Urology.

[59] Jamalirad H., Jajroudi M., Khajehpour B., Gilani M.A.S., Eslami S., Sabbaghian M., Arki H.V. (2025). AI predictive models and advancements in microdissection testicular sperm extraction for non-obstructive azoospermia: A systematic scoping review. Hum. Reprod. Open.

[60] Giulioni C., Phuoc N.H.V., Cayan S. (2025). Ex vivo microscopic testicular sperm extraction at the time of radical orchiectomy in men with nonobstructive azoospermia (NOA): A scoping review. Asian J. Androl..

[61] Pozzi E., Corsini C., Belladelli F., Bertini A., Negri F., Raffo M., Saccà A., Ventimiglia E., Boeri L., Fallara G. (2024). Role of follicle-stimulating hormone, inhibin B, and anti-Müllerian hormone in predicting sperm retrieval from men with non-obstructive azoospermia undergoing microdissection testicular sperm extraction: A systematic review and meta-analysis. Eur. Urol. Open Sci..

[62] Çayan S., Pinggera G.M., Alipour H., Altay B., Shah R., Giulioni C., Mostafa T., Hamoda T., Alarcon D.C.A., Dardmeh F. (2026). The effects of varicocele repair on testicular sperm retrieval, sperm recovery in the ejaculate and clinical pregnancy rates in non-obstructive azoospermic men with clinical varicocele: A systematic review and meta-analysis. World J. Mens. Health.

[63] Boeri L., Dinkelman-Smit M., Jensen C.F.S., Minhas S., Salonia A., European Association of Urology Guidelines Panel on Sexual and Reproductive Health (2026). Varicocele treatment in azoospermia before artificial reproductive techniques: Critical considerations by the EAU Guidelines Panel on Sexual and Reproductive Health. Eur. Urol..

[64] Zhang L., Li Y., Zhang L., Liang Y., Zhuo Y., Huang J. (2026). Testicular shear wave elastography in varicocele: Measurement strategy and clinical application. Basic Clin. Androl..

[65] Giulioni C., Falsetti F., Maurizi V., Del Giudice F., Crocetto F., Imbimbo C., Cafarelli A. (2025). The impact of heavy metals exposure on male fertility: A scoping review of human studies. J. Basic Clin. Physiol. Pharmacol..

[66] Agarwal A., Farkouh A., Saleh R., Hamoda T.A.-A.A.-M., Salvio G., Boitrelle F., Harraz A.M., Ghayda R.A., Kavoussi P., Gül M. (2024). Technical Aspects and Clinical Limitations of Sperm DNA Fragmentation Testing in Male Infertility: A Global Survey, Current Guidelines, and Expert Recommendations. World J. Mens. Health.

[67] Tharakan T., Bettocchi C., Carvalho J., Corona G., Jones T.H., Kadioglu A., Salamanca J.I.M., Serefoglu E.C., Verze P., Salonia A. (2022). European Association of Urology Guidelines Panel on Male Sexual and Reproductive Health: A clinical consultation guide on the indications for performing sperm DNA fragmentation testing in men with infertility and testicular sperm extraction in nonazoospermic men. Eur. Urol. Focus.

[68] Practice Committee of the American Society for Reproductive Medicine (2013). The clinical utility of sperm DNA integrity testing: A guideline. Fertil. Steril..

[69] Çayan S., Farkouh A., Agarwal A., Atmoko W., Wyns C., Arafa M., Zini A., Shah R., Alipour H., Chung E. (2026). Global Andrology Forum Clinical Guidelines on the Relevance of Sperm DNA Fragmentation in Reproductive Medicine. World J. Mens. Health.

[70] Farkouh A., Agarwal A., Hamoda T.A.-A.A.-M., Kavoussi P., Saleh R., Zini A., Arafa M., Harraz A.M., Gul M., Karthikeyan V.S. (2023). Controversy and Consensus on the Management of Elevated Sperm DNA Fragmentation in Male Infertility: A Global Survey, Current Guidelines, and Expert Recommendations. World J. Mens. Health.

[71] Sakkas D., Alvarez J.G. (2010). Sperm DNA fragmentation: Mechanisms of origin, impact on reproductive outcome, and analysis. Fertil. Steril..

[72] Agarwal A., Mostafa T., Al Hashimi M., Çayan S., Pescatori E., Singh K., Şahin B., Zini A., Chung E., Arafa M. (2026). Global Andrology Forum Clinical Guidelines on Antioxidant Use in the Treatment of Male Infertility. World J. Mens. Health.

[73] Bender R., Friede T., Koch A., Kuss O., Schlattmann P., Schwarzer G., Skipka G. (2018). Methods for evidence synthesis in the case of very few studies. Res. Synth. Methods.

[74] Fallara G., Pozzi E., Belladelli F., Boeri L., Capogrosso P., Corona G., D’ARma A., Alfano M., Montorsi F., Salonia A. (2024). A systematic review and meta-analysis on the impact of infertility on men’s general health. Eur. Urol. Focus.

[75] Lotti F., Maggi M. (2018). Sexual dysfunction and male infertility. Nat. Rev. Urol..

